# A Phase 1 dose-escalation study to evaluate safety, pharmacokinetics and pharmacodynamics of AsiDNA, a first-in-class DNA repair inhibitor, administered intravenously in patients with advanced solid tumours

**DOI:** 10.1038/s41416-020-01028-8

**Published:** 2020-08-25

**Authors:** Christophe Le Tourneau, Jean-Pierre Delord, Nuria Kotecki, Edith Borcoman, Carlos Gomez-Roca, Ségolène Hescot, Christiane Jungels, Anne Vincent-Salomon, Vincent Cockenpot, Lauriane Eberst, Audrey Molé, Wael Jdey, Françoise Bono, Véronique Trochon-Joseph, Hélène Toussaint, Christelle Zandanel, Olga Adamiec, Olivier de Beaumont, Philippe Alexandre Cassier

**Affiliations:** 1 Department of Drug Development and Innovation (D3i), Institut Curie, Paris and Saint-Cloud, France; 2 Institut Claudius Régaud, IUCT-Oncopole, Toulouse, France; 3 Medical Oncology Clinic, Institut Jules Bordet, Brussels, Belgium; 4 Department of Tumor Biology, Institut Curie, Paris, France; 5 Medical Oncology, Centre Léon Bérard, Lyon, France; 6 Clinical Department, Onxeo, Paris, France; 7 Scientific Department, Onxeo, Paris, France

**Keywords:** Targeted therapies, Oncology

## Abstract

**Background:**

AsiDNA, a first-in-class oligonucleotide-mimicking double-stranded DNA breaks, acts as a decoy agonist to DNA damage response in tumour cells. It also activates DNA-dependent protein kinase and poly (adenosine diphosphate [ADP]-ribose) polymerase enzymes that induce phosphorylation of H2AX and protein PARylation.

**Methods:**

The aim of this Phase 1 study was to determine dose-limiting toxicities (DLTs), maximum tolerated dose (MTD), safety and pharmacokinetics/pharmacodynamics of AsiDNA administered daily for 3 days in the first week then weekly thereafter. Twenty-two patients with advanced solid tumours were enrolled in 5 dose levels: 200, 400, 600, 900, and 1300 mg, using a 3 + 3 design.

**Results:**

The MTD was not reached. IV AsiDNA was safe. Two DLTs (grade 4 and grade 3 hepatic enzymes increased at 900 and 1300 mg), and two related SAE at 900 mg (grade 3 hypotension and grade 4 hepatic enzymes increased) were reported. AsiDNA PK increased proportionally with dose. A robust activation of DNA-PK by a significant posttreatment increase of γH2AX was evidenced in tumour biopsies.

**Conclusion:**

The dose of 600 mg was identified as the optimal dose for further clinical development.

**Clinical trial registration:**

Clinical trial registration (NCT number): NCT03579628.

## Background

Drug resistance during anticancer therapies is observed in all types of tumours. One of the mechanisms of tumour resistance to anticancer therapy, especially in relapsed tumours with high genetic instability is the enhanced DNA repair activities on DNA double-strand breaks (DSBs).^1–3^ Therefore, there is a high unmet medical need to develop innovative approaches to prevent or reverse this type of resistance.

The development of poly (ADP-ribose) polymerase inhibitors (PARPi) to treat patients with homologous recombination (HR)-deficient tumours (harbouring *BRCA1* or *BRCA2* mutations) is the first example of exploiting the DNA repair defects^4–6^ to treat cancer. However, despite the good safety and efficacy of these inhibitors, they target only a small population with a specific genetic defect, and tumours can easily acquire resistance to treatment by restoring the HR repair, hyperactivating the non-homologous-end-joining repair or over-expressing the P-glycoprotein drug efflux pumps.^7^ Hence, the hurdles faced by these well-tailored therapies raised the issue of developing a new family of DNA repair inhibitors with a wider spectrum of action to avoid the emergence of resistance.

AsiDNA (INN: etidaligide—previous names DT01 or coDbait) is a first-in-class DNA repair inhibitor designed to prevent the repair of DNA damage in tumour cells. AsiDNA is a double-stranded DNA molecule consisting of 32 base pairs with a linker on one side, to stabilise the double-strand structure, and a free end on the other to mimic a DSB.^8^ A cholesterol vector linked to its 5′-end facilitates the penetration of the molecule into the cells. The mechanism of action of AsiDNA is through the trapping and the hyperactivation of DNA-dependent protein kinase (DNA-PK) proteins. This hyperactivation of DNA-PK leads to downstream targets of phosphorylation, such as histone H2AX, leading to a pan-nuclear γH2AX signal.^9^ This false damage signalling inhibits the recognition of genomic DSBs and the recruitment of repair proteins for efficient repair.^8,9^ AsiDNA also acts as a lure for PARP1 proteins, which are also hyperactivated, leading to the sequestration of proteins acting in the same pathway (XRCC1, PCNA).^10^ This overall hyperactivation of DNA-PK and PARP proteins allows quantifying the activity of AsiDNA in cells and tumours. Thus DNA-PK targets such as the pan-nuclear γH2AX signal and pHSP90 (phosphorylated form of HSP90 proteins), as well as the global protein PARylation induced by the hyperactivation of PARP, constitute activity biomarkers of AsiDNA that could be analysed.

Preclinical proofs of AsiDNA’s activity have been obtained in various tumour models with a high basal level of DNA lesions, due to downregulated DNA repair pathways, and especially DSB repair pathways (uveal and cutaneous melanoma, glioblastoma, colorectal cancer, breast cancer, liver cancer, lung cancer, cervix cancer and head and neck cancer).^11,12^ AsiDNA’s effect does not appear to be specific to the tumour type. The pharmacological effect has been confirmed with different routes of administration (intratumoural [IT], intraperitoneal, intravenous [IV], subcutaneous [SC], intra-arterial) and with different treatment modalities: monotherapy, combination with radiotherapy (RT) and other locoregional treatment (i.e., radiofrequency ablation, transcatheter arterial chemoembolisation), various chemotherapy [CT] and PARPi.^13–17^ AsiDNA was shown to induce cumulative antitumour activity with low probability of acquired resistance in preclinical models.^18^ In the clinic, the drug was first investigated in 23 patients with skin metastases of melanoma in a Phase 1 dose-escalation study,^19^ which evaluated the safety and pharmacokinetics (PK) of AsiDNA administered by SC (IT/peri-tumoural) injections in combination with palliative RT. This first-in-human study of AsiDNA was conducted as a proof of concept of the ability of the drug to sensitise and improve RT response rate (estimated about 10% of complete response rate in melanoma) based on preclinical data showing an AsiDNA-induced radio-sensitisation in human melanoma xenografted models.^20^ Twenty-one patients were evaluable for efficacy on 76 lesions. Objective responses were observed in 45 lesions (59%), including 23 complete responses (30%). The PK analyses suggested a systemic passage of AsiDNA.^20^ The most common related adverse events (AEs) reported in this study were grade 1 and 2 local site injection reactions (pain and erythema) occurring in 12 (52%) and 7 (30%) patients, respectively.^20^

Here we present the results of the first Phase 1 trial evaluating AsiDNA by IV route in patients with various advanced solid tumours (The DRIIV-1 [DNA Repair Inhibitor-administered IntraVenously] study). The purpose of this study was to assess, safety, PK and pharmacodynamics (biomarkers of activity) of AsiDNA administered IV as single agent, in order to identify dose-limiting toxicities (DLTs) and maximal tolerated dose (MTD) and establish the recommended dose (RD) for further clinical development.

## Methods

### Patient selection

This was an open-label, dose-escalation Phase 1 trial. The study was conducted at 4 centres in France and Belgium (Institut Curie—Paris, Institut Claudius Régaud IUCT—Oncopôle Toulouse, Centre Léon Bérard—Lyon and Institut Jules Bordet—Brussels). The study received approval from the Central Ethics Committees of “Rennes Ouest V” in France and “Central Ethics Committee of “Institut Jules Bordet”, Brussels in Belgium and conducted in accordance with the ICH (Harmonized Tripartite Guidelines for Good Clinical Practice) and the Declaration of Helsinki. Written informed consent was obtained from all participants. The trial was registered at ClinicalTrials.gov (number NCT03579628) and assigned its Eudract number (2017-000088-34).

Patients with advanced solid tumours who failed or for whom no standard treatment option existed were eligible for this study. Prior anticancer therapies were allowed provided that an interval of at least 21 days has elapsed between the last therapy and the first study drug administration. Main other eligibility criteria included: age ≥18 years, Eastern Cooperative Oncology Group (ECOG) scale performance status (PS) of 0 or 1, life expectancy of at least 3 months, adequate haematological (absolute neutrophil count ≥1.5 × 10^9^/L, haemoglobin level ≥9 g/dL, platelet count ≥100 × 10^9^/L), liver (total bilirubin level ≤ upper limit normal [ULN], aspartate transaminase [AST] and alanine transaminase [ALT] ≤ 1.5 × ULN, in case of liver metastases AST/ALT ≤ 3 × ULN) and renal function tests (calculated glomerular filtration rate [GFR] ≥60 mL/min/1.73 m^2^ per Chronic Kidney Disease Epidemiology Collaboration [CKD-EPI] formula), and measurable or assessable disease. Main exclusion criteria included any of the following: symptomatic or active brain metastases; significant concomitant systemic disorders incompatible with the study (i.e. chronic liver diseases; uncontrolled metabolic disorders, positive HIV, hepatitis B or C; uncontrolled congestive heart failure defined as New York Heart Association class III or IV, uncontrolled hypertension, unstable heart disease [e.g. coronary artery disease with unstable angina or myocardial infarction within 6 months before study treatment administration] or any significant electrocardiogram (ECG) abnormalities defined as any cardiac dysrhythmia] >grade 2 i.e. significant ventricular arrhythmia as persistent ventricular tachycardia and/or ventricular fibrillation; severe conduction disorders as atrioventricular block 2 and 3, sinoatrial block or baseline QT/QTc interval >480 ms). Patients with abnormal urinalysis at screening (i.e. haematuria >1+ on dipstick, confirmed on retest, in the absence of documented urinary infection, and/or proteinuria >1+ on dipstick, confirmed on retest, with further confirmation by quantitative total urine protein >1.0 g/24 h) were not eligible. Pregnant and breastfeeding women were excluded from the study.

### Treatment plan and study design

AsiDNA (Onxeo, Paris, France) was supplied with no cost to the study participants in labelled sterile lyophilised powder for solution for infusion containing 110 mg AsiDNA, packaged in a 20-mL glass vial sealed with an aluminium cap fitted with a polypropylene disk. The vials were to be stored in a freezer (at −20 ± 5 °C) until preparation. AsiDNA was administered IV by electric pump as a 1-h IV infusion. AsiDNA was given daily for 3 days in the first week, then weekly thereafter. A treatment cycle consisted of 21 days of treatment period. Patients were planned to receive AsiDNA until disease progression, unacceptable toxicity or patient’s refusal to continue, whichever occurred first.

Patients were treated with ascending doses of AsiDNA according to a “3 + 3 design” using a sentinel patient at each cohort; the first patient of each cohort was monitored for 1 week (i.e. 7 days after the first dose) before the next two patients of the same cohort could be enrolled. Six dose levels (DLs) were planned (DL1–DL6): 200, 400, 600, 900, 1300, and 1800 mg. No intra-patient dose escalation was allowed. Three patients were treated at each DL and if no cycle-1 DLTs were observed, three additional patients were to be treated at the next DL. If one of three initial patients experienced a DLT at any given DL, three additional patients were to be treated at that same DL. If a DLT occurred in at least two patients at any DL, then dose escalation was halted, and the next three patients enrolled onto that treatment cohort were to be treated at the next lower DL. The dose at which unacceptable toxicity was seen in at least 2/3 or 2/6 patients was considered as the unacceptable dose. The MTD was considered as the dose immediately below this dose or the highest tested dose if no DLT was observed at this dose. DLT was defined as any of the following adverse events occurring during cycle 1: grade 4 neutropenia lasting ≥7 days, febrile neutropenia, grade 4 thrombocytopenia or grade 3 thrombocytopenia associated with bleeding, any drug-related non-haematological toxicity grade ≥3 (except alopecia, fatigue, nausea and vomiting adequately treated with anti-emetic treatment and non-clinically significant laboratory value abnormalities). A Data Safety Monitoring board (DSMB) oversaw the study conduct and reviewed all DLTs and overall safety and PK data in order to support the selection of the optimal RD. Prior approval from the DSMB had also to be granted before dose escalation.

### Study work-up

For each cycle of AsiDNA administration, vital signs (body weight, temperature, blood pressure and heart rate (HR), adverse events (AEs) and serious AEs (SAEs), physical examination, ECOG PS and concomitant medications were collected on a regular basis). Weekly laboratory evaluations included full blood count performed before each cycle with differentials, complete biochemistry including sodium, calcium, potassium, phosphate, chloride, fasting glucose, serum albumin, total protein, lactate dehydrogenase, alkaline phosphatase, gamma-glutamyl transferase, AST, ALT, total bilirubin, indirect bilirubin, serum urea level, serum creatinine, calculated GFR (CKD-EPI), cystatin C, immunoglobulin G (IgG), IgA, IgM and C-reactive protein and coagulation tests (activated partial thromboplastin time and international normalized ratio).

Based on minor AEs observed with AsiDNA in non-clinical toxicology studies in Cynomolgus monkeys treated with AsiDNA by IV route, mainly histological vascular changes (sub-acute and chronic inflammation in vessels wall of the kidneys of the animals), exploratory renal biomarkers were monitored in all patients to mitigate this potential risk. Three predictive urine biomarkers associated with potential nephrotoxicity were collected in patients’ urine samples: b2-microglobulin as an acute glomerular alteration biomarker, microalbumin, and kidney injury molecule-1 (KIM-1) as acute kidney tubular alteration biomarkers at cycle 1 on D1, D3, D8 and D15, within 4 h after the end of AsiDNA infusion, on D1 of each subsequent cycle after the end of AsiDNA infusion (within 4 h after the end of AsiDNA infusion) and at the end of the study treatment. Urinalysis for specific gravity, pH, protein, blood, glucose, ketones, bilirubin, urobilinogen, leucocytes and nitrites on fresh urine sample were also done on a weekly basis; in case of presence of proteinuria ≥+2 on dipstick, dipstick test was repeated and a quantitative 24 h urine protein and protein-to-creatinine ratio were done to confirm proteinuria.

To evaluate inflammatory response, whole blood samples were collected at pre-dose and 6 h (±1 h) and 23 h (±1 h) post-dose at cycle 1 on days 1 and 3. Cytokine (interleukins (ILs; IL-2, IL-6 and IL-12p70) and interferons (IFNs; IFNa2 and IFNg)) and chemokine (IP-10 (IFNg inducible protein or CXCL10) and MCP-1 (monocyte chemoattractant protein 1 or CCL2)) plasma levels were quantified using a magnetic bead-based multiplex immunoassay and C3a complement factor plasma levels were determined using an enzyme-linked immunosorbent assay (ELISA) assay.

Lipid profile (total cholesterol, high-density lipoprotein–cholesterol, low-density lipoprotein–cholesterol, triglycerides) was monitored before each cycle given the cholesterol-conjugated 32-base pair double-stranded DNA portion of AsiDNA. For all patients, ECGs were performed before each cycle; in addition, 4 triplicates 12-lead ECG were recorded for central reading (Cardiabase, Nancy, France); at baseline (prior to the first dose), at cycle 1 on D1 (at the end of the infusion) and on D3 (at pre-dose and end of the infusion). The first tumour evaluation was done after 2 cycles (i.e. [D37–D42], then every 2 cycles (i.e. every 6 weeks) using RECIST 1.1 if there is at least one measurable lesion at baseline or according to the clinical practice (i.e. clinical examination, biomarkers/specific tumour guidelines) if there was no measurable lesion at baseline.

### Statistical methods

No formal statistical hypotheses were set up for the sample size calculation. Descriptive statistics were performed using the SAS^®^ software version 9.4. All clinical and laboratory data were summarised by DL and overall. All safety analyses were performed on all enrolled and treated patients. AEs were graded using the National Cancer Institute [NCI] Common Terminology Criteria for Adverse Events scale, version 4.03. All treatment-emergent AEs (TEAEs) and related TEAEs that was considered as probably, possibly or definitely related to AsiDNA are summarised in tables by DL and overall.

### PK and bioanalytical methods

Blood samples were collected from 22 patients during treatment at cycle 1 D1 and D3 at pre-dose, 30 min (± 5 min), 55 min (± 5 min) after start of infusion and 15 min (± 5 min), 30 min (± 5 min), 1 h (± 10 min), 3 h (± 10 min), 6 h (± 1 h) and 23 h (± 1 h) after the end of infusion. AsiDNA was quantified in plasma using a GLP validated hybridisation ELISA assay with a low limit of quantification of 25.0 ng/mL. After the first dose and the third dose of cycle 1 of AsiDNA, the following PK parameters were calculated, from AsiDNA concentration–time data using the SAS 9.4 software: *C*_max_, *t*_max_, area under the curve (AUC)_0–last_, AUC_0–∞_, *t*_1/2_, and clearance (CL) by Calvagone (Liergues).

### Pharmacodynamics

To confirm that AsiDNA reached patient tumours and was up taken by tumour cells, we analysed target engagement and AsiDNA-induced false damage signalling (pan-nuclear phosphorylation of H2AX and Hsp90, so called γH2AX and pHsp90, respectively) before (Pre) and after (Post) treatment. Cell proliferation marker (Ki67) was also analysed on the tumour samples. Two sequential biopsies were taken from the patients: one fresh tumour sample within 4 weeks prior to the first AsiDNA dose, to define biomarkers basal levels, and a second tumour biopsy taken 24 h after D1 cycle 2. Biopsies were formalin fixed, paraffin embedded and cut for DAB-labelling immunohistochemistry (IHC) biomarker analysis by the pathology department of Institut Curie (Paris). Biopsies were fixed with 4% paraformaldehyde and paraffin embedded. Biopsy sections were then permeabilised and IHC was performed using mouse anti-γH2AX monoclonal antibody (clone JBW—Merck, 1/200), rabbit anti-phosphoHSP90 (Cell Signaling, 1/200) and mouse anti-Ki67 antibody (Dako Agilent, 1/200). A blinded histological quantification of percentage of tumour cells positive for each staining was performed by an experienced pathologist (Pathology department, Institut Curie, Paris).

## Results

### Patients and drug exposure

A total of 22 patients were enrolled and treated and included in the analysis. The study population comprised of females in 59.1%, mean age of the study population was 58.8 years (range: 38 to 78), and main tumour types were colorectal (5/22; 22.7%) followed by breast cancer and sarcoma (3/22; 13.6%, each). Most (19/22; 86.4%) patients had ≥3 prior lines of anticancer therapies (Table 1).

**Table 1:** Patient demographics and baseline characteristics.

	Total (*N* = 22)
Age (years)	
Mean (SD)	58.8 (9.7)
Range	38.0–78.0
Sex (*n*; %)	
Female	13 (59.1)
Male	9 (40.9)
ECOG PS (*n*; %)	
0—Fully active	14 (63.6)
1—Restricted in physically strenuous activity	8 (36.4)
Tumour type (*n*; %)	
Colorectal	5 (22.7)
Breast	3 (13.6)
Sarcoma (of the thigh, uterus and stomach)	3 (13.6)
Uterus	2 (9.1)
Pancreas	2 (9.1)
Liver	1 (4.5)
Oesophagus	1 (4.5)
Ovary	1 (4.5)
Small intestine	1 (4.5)
Stomach	1 (4.5)
Testicles	1 (4.5)
Vater ampulla	1 (4.5)
Number of metastatic sites	
Median (range)	2 (0–5)
Prior lines of anticancer therapy (*n*; %)	
1	1 (4.5)
2	2 (9.1)
≥3	19 (86.4)

All patients received at least one cycle of study treatment for a total of 164 AsiDNA doses given by IV infusions at the ascending doses of 200 mg (*n* = 3), 400 mg (*n* = 4), 600 mg (*n* = 3), 900 mg (*n* = 6) and 1300 mg (*n* = 6) with a total number of AsiDNA doses administered during the study of 27, 26, 36, 42 and 33, respectively.

### Dose-limiting toxicities

Twenty-one out of the 22 treated patients were evaluable for DLT determination and 2 DLTs occurred during the study (Table 2). No DLTs or drug-related SAEs were observed in the first 9 evaluable patients treated at 200 mg, 400 mg and 600 mg. At 900 mg (DL4), one patient developed a DLT (grade 4 hepatic enzymes increased); this 61-year-old female diagnosed with gastric adenocarcinoma and liver and peritoneum metastases had liver enzymes (AST/ALT) increased to 664 UI/L and 425 UI/L, respectively, 8 days after the first dose. Baseline AST/ALT were 52 UI/L and 43 UI/L, respectively. After 2 days, liver enzymes decreased in severity to grade 2 (AST at 123 UI/L, ALT at 196 UI/L), and the patient was discharged from hospital and withdrawn from the study. Five days after discharge from hospital, liver enzymes increased to grade 3 (AST at 482 UI/L, ALT at 466 UI/L) associated with a bilirubin increase to grade 2 (total bilirubin at 2.2 mg/dL). The patient was re-hospitalised for further investigations. Liver imaging showed diffuse hepatic metastatic infiltration to the left lobe and the hepatic hilar region with moderate dilation of the intrahepatic biliary tracts, thrombosis of the left portal vein and the median and left sub-hepatic vein with moderate ascites. Liver biopsy showed an acute inflammatory alteration in healthy liver tissue and in metastatic tissue. This event was considered by the investigator as serious and possibly related to AsiDNA given the temporal relationship to dosing. The patient died 1 month after event onset, due to disease progression; liver enzymes never came back to baseline. At 1300 mg (DL5), one patient, a 48-year-old female with metastatic breast cancer, experienced a DLT (grade 3 hepatic enzymes increased); the patient reported an aggravation of pre-existing hepatic cytolysis to grade 3 (AST at 158 IU/L, ALT at 403 IU/L), at day 8 after the first dose. In her medical history, hepatic cytolysis (grade 2) was present prior to study entry and was likely due to liver metastases. The event was considered as serious and the patient was withdrawn from the study. The investigator considered initially the event as possibly related to AsiDNA, then changed afterward his assessment to not related to AsiDNA due to the concomitant underlying disease progression. However, considering the temporal relationship between the occurrence of the event and AsiDNA administration, the DSMB maintained the causal relationship of the event to AsiDNA. The cohort was then expanded to three additional patients, without any further toxicity.

**Table 2:** Planned dose escalation, actual enrolment and dose-limiting toxicities (DLTs).

Dose level	Dose escalation (mg)	No. of patients treated	No. of patients evaluable for DLT	No. of patients with DLT	DLT type
DL1	200	3	3	0/3	—
DL2	400	4^a^	3	0/3	—
DL3	600	3	3	0/3	—
DL4	900	6	6	1/6	G4 hepatic enzymes increased
DL5	1300	6	6	1/6	G3 hepatic enzymes increased

*DL* dose level, *DLT* dose-limiting toxicity, *G* grade.

^a^One patient not evaluable for DLT (withdrawn from the study before the end of DLT evaluation period due to a non-serious adverse event related to underlying disease progression).

### TEAEs and biological evaluation

TEAEs were reported in 21 (95.5%) patients for a total of 198 events, of which 63 (31.8%) in 16 (72.7%) patients were related to study drug. Most (173/198; 87.7%) TEAEs were of mild-to-moderate intensity and 12.6% (25/198) were of grade ≥3 intensity; among them 3 TEAEs grade ≥3 were related to AsiDNA; these were grade 3 hypophosphatemia, grade 3 hypotension and grade 4 hepatic enzyme increased, all occurred at 900 mg (*n* = 2). No related TEAE ≥grade 3 was reported at 200, 400 and 600 mg. No related TEAE ≥grade 3 was reported at 1300 mg either. No grade 5 TEAE was reported during the study treatment. Four (18.2%) patients discontinued study treatment due to non-fatal AEs (one patient at 400 mg, one patient at 900 mg and 2 patients at 1300 mg). Two of the TEAEs that led to study withdrawal were considered as serious and fulfilled the criteria of a DLT (as described above). Other TEAEs that led to treatment discontinuation included grade 2 creatinine increase and grade 2 bilirubin increase in one patient and grade 3 AST increase in one patient. A total of 9 events (in 7 patients) were considered as serious, of which only 2 were related to study drug (grade 4 hepatic enzymes increased [DLT] and grade 3 hypotension), both occurred at DL4 (900 mg). Most frequently reported related TEAEs were asthenia and tinnitus reported in 4 (18.2%) patients each, followed by AST enzymes increased and hypercholesterolaemia in 3 (13.6%) patients each and fatigue, ALT enzymes increased, abdominal pain, diarrhoea, nausea and anaemia in 2 (9.1%) patients each (Table 3).

**Table 3:** Treatment-emergent adverse events (TEAEs) related to AsiDNA reported in more than one patient (*N* = 22).

System organ class/preferred term	All grades		Grade 1		Grade 2		Grade 3		Grade 4	
	*N* (%)	*E*	*N* (%)	*E*	*N* (%)	*E*	*N* (%)	*E*	*N* (%)	*E*
At least one related TEAE	16 (72.7)	63	13 (59.1)	44	9 (40.9)	16	2 (9.1)	2	1 (4.5)	1
Gastrointestinal disorders	6 (27.3)	9	6 (27.3)	8	1 (4.5)	1	—	0	—	0
Abdominal pain	2 (9.1)	2	2 (9.1)	2	—	0	—	0	—	0
Diarrhoea	2 (9.1)	2	2 (9.1)	2	—	0	—	0	—	0
Nausea	2 (9.1)	2	2 (9.1)	2	—	0	—	0	—	0
General disorders and administration	6 (27.3)	12	5 (22.7)	8	3 (13.6)	4	—	0	—	0
Site conditions										
Asthenia	4 (18.2)	5	3 (13.6)	3	2 (9.1)	2	—	0	—	0
Fatigue	2 (9.1)	5	2 (9.1)	3	1 (4.5)	2	—	0	—	0
Investigations	5 (22.7)	9	4 (18.2)	6	2 (9.1)	2	—	0	1 (4.5)	1
Aspartate aminotransferase increased	3 (13.6)	4	3 (13.6)	3	1 (4.5)	1	—	0	—	0
Alanine aminotransferase increased	2 (9.1)	2	2 (9.1)	2	—	0	—	0	—	0
Metabolism and nutrition disorders	5 (22.7)	7	2 (9.1)	4	2 (9.1)	2	1 (4.5)	1	—	0
Hypercholesterolaemia	2 (9.1)	3	2 (9.1)	3	—	0	—	0	—	0
Ear and labyrinth disorders	4 (18.2)	4	4 (18.2)	4	—	0	—	0	—	0
Tinnitus	4 (18.2)	4	4 (18.2)	4	—	0	—	0	—	0
Blood and lymphatic system disorders	3 (13.6)	4	2 (9.1)	3	1 (4.5)	1	—	0	—	0
Anaemia	2 (9.1)	2	1 (4.5)	1	1 (4.5)	1	—	0	—	0

NB: Not in the Table: one G4 hepatic enzymes increased in one patient at 900 mg related to AsiDNA, which was a DLT, and one G3 hypophosphatemia related to AsiDNA in one patient.

*E* event; *N* number of patients.

One patient, a 64-year-old male treated at DL2 (400 mg) presented with a non-serious renal event consistent with a chronic kidney disease of grade 2, judged by the investigator as possibly related to AsiDNA. On C3D8, the patient reported haematuria grade 1, which resolved the same day, followed by polyuria grade 1 and an increase in serum creatinine to grade 1 (120 µmol/L versus 88 at baseline). Calculated GFR was 56 mL/min (versus 76 mL/min at baseline) and KIM-1 was found to be slightly increased (above the normal range of 2146 pg/mL). All other investigations (urinary echography, renal biopsy and urinary magnetic resonance imaging were normal). The patient was withdrawn from the study. The events resolved 2 months later. No significant sustained increase of any of the three renal biomarkers monitored was observed during treatment in any of the other patients.

Overall, AsiDNA induced a transient inflammatory response in some patients independently of the dose. Upregulations on cytokines and chemokines were limited and not sustainable over the time and were variable from one patient to another at the same DL (data not shown). AsiDNA did not induce any modification of the level of plasmatic complement factor (C3a) after a single or three consecutive IV (1-h infusion) administrations of AsiDNA. A slight but not clinically significant change in laboratory parameters were reported as possibly related to AsiDNA; these were grade 2 hypomagnesaemia in one patient (1 event), grade 3 hypophosphataemia in 1 patient (1 event), and grade 1 hypercholesterolaemia in 2 patients (3 events) treated at 600 and 900 mg.

### Triplicate 12 lead-ECG evaluation

ECG records from a total of 22 subjects were analysed. One subject (600 mg) was excluded from the analysis because only paper ECGs were recorded at pre-dose on day 1 and a single digital ECG at day 3 pre-dose was received. There were no relevant changes for the ECG intervals of interest (HR, QRS, PR) after IV administration of AsiDNA. The categorical and morphological analyses did not reveal any treatment-related effect. The concentration–response analysis did not indicate that there was an effect on QTcF or HR with increasing concentration of AsiDNA. At the geometric mean *C*_max_ of all doses, a similar small increase of about 2 ms was predicted with 90% confidence interval remaining below the regulatory threshold of 10 ms.

### PK results

**Fig. 1:**
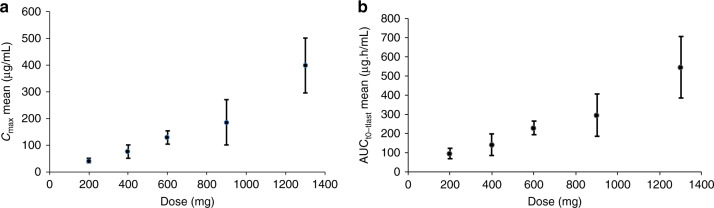
Dose–response exposures of AsiDNA after IV (1-h infusion) administration in patients across the tested doses, DL1 (200 mg), DL2, (400 mg), DL3 (600 mg), DL4 (900 mg) to DL5 (1300 mg). *C*_max_ mean (**a**) and AUC_t0-tlast_ (**b**) are represented after the first and the third administration from 3 patients at 200 and 600 mg cohort, 4 patients at 400 mg cohort and 6 patients at 900 mg and 6 patients at 1300 mg cohort.

After IV administration (1-h infusion) of AsiDNA, the peak of AsiDNA concentration (*C*_max_) was observed in most of the patients just before the end of the infusion. After the end of the infusion, it appeared that AsiDNA is multi-phasic, with a terminal half-life of about 3–5 h with a minimal value of 1.54 and a maximal value of 9.71 h. Total CL was around 2.50 L/h (42 mL/min) with a minimal value of 1.55 and a maximal value of 5.55 L/h (25.8 and 92.5 mL/min, respectively). Median of PK parameters from all cohorts are presented in Table 4. No difference was observed in AsiDNA PK after administration at days 1 and 3 of cycle 1 with *R*_max_ and *R*_AUC_ close to 1. AsiDNA *C*_max_ and AUC increased proportionally and consistently with dose with no difference between day 1 and day 3 in the dose range of 200–1300 mg, as shown in Fig. 1.

**Table 4:** AsiDNA pharmacokinetic parameters after the first and the third administration of AsiDNA by IV (1-h infusion) route from DL1 to DL5.

AsiDNA cycle 1 day 1					AsiDNA cycle 1 day 3			
Median [minimum–maximum]					Median [minimum–maximum]			
Dose (mg)	*C*_max_ (ng/mL)	*t*_max_ (h)	AUC_last_ (ng.h/mL)	CL (L/h)	*C*_max_ (ng/mL)	*t*_max_ (h)	AUC_last_ (ng.h/mL)	CL (L/h)
200	42,123 [38,449–55,836]	1.25 [0.917–1.35]	96,596 [82,078–128,652]	1.98 [1.55–2.40]	36,035 [32,693–45,408]	0.917 [0.917–1.20]	94,118 [55,170–118,842]	1.68 [1.68–1.68]
400	71,471 [38,480–119,060]	0.917 [0.500–1.22]	110781 [89,445–236,239]	3.69 [1.67–4.44]	75,442 [64,639–99,393]	0.917 [0.917–0.917]	127,462 [102,909–220,713]	3.14 [1.81–3.89]
600	137,342 [82,802–143,747]	0.917 [0.467–0.950]	254,672 [162,437–256,918]	2.35 [2.32–3.65]	141,138 [123,206–147,954]	0.950 [0.933–0.950]	237,224 [232,150–238,259]	2.53 [2.52–2.58]
900	150835 [91,279–393,534]	1.01 [0.500–1.28]	311,105 [161,129–520,430]	2.90 [1.73–5.55]	179,338 [126,930–285,174]	0.983 [0.917–1.25]	246,037 [204,358–385,973]	3.66 [2.33–4.40]
1300	375847 [338,946–618,618]	0.934 [0.567–1.25]	515,015 [382,229–822,747]	2.51 [1.58–3.39]	404,071 [231,811–512,882]	0.942 [0.917–0.967]	498,370 [298,665–684,756]	2.63 [1.90–4.35]

Blood samples for PK analysis were collected from 22 patients. Of note, for 2 subjects who received AsiDNA 200 mg, samples were missing at 23 h after the third infusion, and 1 patient receiving AsiDNA 900 mg had issues during the third infusion, therefore this patient was not included in the analysis.

### Biomarkers of activity in tumours tissue

A total of 11 paired tumour biopsies were collected; no posttreatment biopsy was available at 200 mg. Hence, 10 paired biopsies were evaluable for a quantitative change in tumour biomarkers as follows: 200 mg = 0, 400 mg = 2, 600 mg = 2, 900 mg = 4 and 1300 mg = 2.

**Fig. 2:**
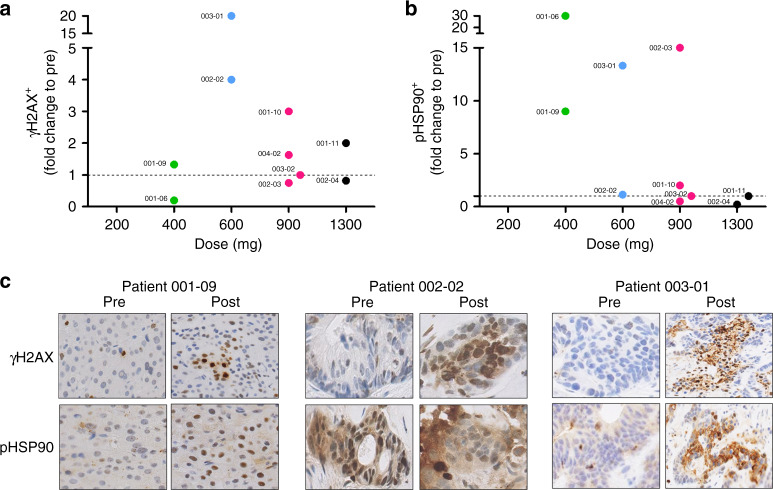
AsiDNA activity biomarkers in tumor samples. **a**, **b** AsiDNA activity biomarkers: γH2AX and pHSP90 positive cells quantification by IHC (Institut Curie). Eleven paired tumour biopsies were collected; no post treatment biopsy was available at the dose of 200 mg. Hence 10 paired biopsies were evaluable for a quantitative change in tumour biomarkers as follows: 200 mg = 0, 400 mg = 2, 600 mg = 2, 900 mg = 4 and 1300 mg = 2. Maximum increase of AsiDNA phosphorylation (γH2AX) is observed at 600 mg. **c** Images from Patient 003-01 tumor biopsies were provided since clear and important biomarker activation was observed with this patient. Other biopsies images showing H2AX staining from different patients are provided.

Positive γH2AX, pHsp90 and Ki67 tumour cells were quantified and results are expressed in percentage of positive cells. Relative levels of expression compared to individual basal level (fold change) are presented in Supplementary Table 1. After one cycle of AsiDNA treatment, for most patients, an increase of the phosphorylation of at least one of AsiDNA activity biomarker (γH2AX and/or pHsp90) was observed. Indeed, 80% of patients are either positive for γH2AX or pHsp90 signals. Measurement of tumour cell proliferation by Ki67 expression revealed a decrease or a stabilisation in 90% of patients after one cycle of AsiDNA treatment. AsiDNA-induced γH2AX signal in patient tumours was observed at all DLs (no tumour tissue at DL1 200 mg) with highest levels observed at DL3, 600 mg with 4–20-fold changes in γH2AX to pre-DLs in the 2/2 patients with analysed paired biopsies (Fig. 2a, b). In Fig. 2c, IHC representative images for patients 001-09 (DL2—400 mg), 002-02 (DL3—600 mg) and 003-01 (DL3—600 mg) showed the specific AsiDNA-induced pan-nuclear staining of γH2AX and pHsp90, as observed in preclinical studies.^8,9,11^

### Antitumoural activity

The best overall response in the 21 evaluable patients was stable disease in 2 patients with metastatic colorectal cancer and treated at 600 mg and progressive disease in the 19 remaining patients. All patients discontinued treatment due to disease progression.

## Discussion

The use of inhibitors of DNA damage signalling and/or repair pathways appears to provide an interesting opportunity for targeting genetic differences between tumour and normal cells.^21^ Introducing small DNA molecules (Dbait) that impair the repair of damaged chromosomes in the therapeutic armament of patients suffering from cancer provides a new method for enhancing the efficiency of standard anticancer therapies, such as RT and/or CT and/or PARPi.

The specificity of AsiDNA mechanism of action in the tumour cells is mainly due to the accumulation of “accidents” during spontaneous tumour growth due to oxidative stress, genomic instability and the impaired cell cycle controls (checkpoints) in tumour cells. Cycle cell progression despite the presence of unrepaired DSBs leads to mitotic catastrophe. AsiDNA breaks DNA repair cycle at the early stage of the process, without requiring any genetic defect (in contrast to PARPi). AsiDNA does not induce any new lesions on chromosomes but amplifies the consequences of spontaneous lesions; it has a specific effect on tumour cells without affecting healthy tissue.^11^

The results of this study of IV AsiDNA show that AsiDNA can be safely administered in patients with advanced solid tumours; the MTD was not reached at the highest dose tested of 1300 mg. AsiDNA was well tolerated in the range of the tested doses (200–1300 mg); most TEAEs were of mild-to-moderate intensity and only 2 patients out of the 21 evaluable patients experienced a DLT, which consisted of elevation of liver enzymes at DL 900 mg (1/6) and 1300 mg (1/6). Although assessed as possibly related to AsiDNA, these events were associated with confounding factors such as liver metastases and underlying disease progression. One patient had a non-serious potential renal toxicity that was compatible with a chronic kidney disease (grade 2), which resolved spontaneously after study drug discontinuation. AsiDNA was not associated with immune or inflammatory response in the range of the tested doses, nor changes in the blood parameters were observed.

IV AsiDNA PK exhibited a linear dose proportionality in the range of the tested doses (from 200 to 1300 mg). AsiDNA peak concentration (*C*_max_) was reached at approximately 1 h after the infusion followed by a multi-phasic decline with a terminal half-life about 3–5 h across doses. No difference was observed in AsiDNA PK after administration at days 1 and 3.

One of the earliest steps in the cellular response to DSBs is the phosphorylation of Ser139 of histone H2AX by the phosphatidylinositol 3-kinases.^22–24^ The appearance of the phosphorylated form of the histone in nuclei, referred to as γH2AX, is often used as an indicator of the presence of DNA DSBs. γH2AX foci formation at DSB sites can recruit other DSB signalling and repair factors such as MDC1, 53BP1 and the MRN complex to the damage site.^25^ Over the past several years, γH2AX expression has been established as a very sensitive surrogate biomarker of DSBs, mostly following radiation—less literature exists about CT.^8,18,26–28^ γH2AX expression remains phosphorylated if the DSBs are not repaired due to defects in the DSB repair machinery. Preclinical data in tumour showed that the AsiDNA-induced false damage signalling of γH2AX, phosphorylated Hsp90 and PARP activation significantly increased between 15 and 48 h after each AsiDNA administration and could be considered as early biomarkers of activity.^29^ In this study, although tumour samples were limited (only 10 paired biopsies were collected among the 22 treated patients), proof of AsiDNA mechanism of action was demonstrated with the increase of the false γH2AX and pHsp90 in the analysed tumour tissues, in particular at DL3 (600 mg) where 4–20-fold changes in γH2AX to pre-DLs were observed. No clear correlation was observed between these pharmacodynamic biomarkers and PK parameters, confirming our preclinical observations, where relevance of H2AX phosphorylation as a surrogate biomarker is clearly dependent on H2AX levels, which highly differ between tumour models, and therefore between patients, making a correlation quite difficult to achieve. In line with this, a correlation could certainly be observed if we implement a dose escalation in the same patient. However, clear signs of biomarker activation were observed posttreatment compared to pretreatment biopsies in 80% of the tested patients, informative of drug uptake into tumours and target engagement, which was the main objective of these biomarker analysis. Another important point that could be highlighted is that AsiDNA, in contrast with other anticancer treatments, is not an inhibitor of a specific target involved in tumour growth, but it is a decoy agonist (AsiDNA binds and activates in particular DNA-PK and redirects it away from sites of tumour DNA damage). Taking into account this very unique mechanism of action, it is certainly impossible to obtain a strict correlation between the dose of AsiDNA and the tumour activity biomarker content. Indeed, increasing concentrations of AsiDNA will increase the biological response until there are no free DNA-PK for AsiDNA to bind or H2AX to be phosphorylated. At this stage, a maximal response is reached that could not be increased. These two parameters: free DNA-PK and H2AX content will be highly different from one patient to another (as discussed before). However, based on our results, and as previously reported,^30^ we could conclude that at a dose of 600 mg the maximal biological response is reached.

Based on these results and following an in-depth analysis of all safety, PK and PD data, the dose 1800 mg was not tested, and the dose 600 mg was selected as the optimal active biological dose for further clinical development of AsiDNA in particular, in combination with DNA-damaging CT and/or PARPi. The expected benefit of the combination of AsiDNA with CT is based on synergistic effect showed in non-clinical studies.^13–15^ In a study using NCI-H446 cells, initially sensitive to carboplatin treated with or without AsiDNA, cell populations treated with both carboplatin (2.5 µM) (high dose corresponding to the IC90) and AsiDNA (low dose—2.5 µM) remained very sensitive to the drugs, while carboplatin resistance was observed in all cell populations treated with carboplatin alone after repeated cycles, demonstrating that AsiDNA, at a low sub-active dose, abrogated the emergence of acquired resistance to carboplatin.^15^ In fact, by abrogating the DNA repair machinery, AsiDNA sensitises tumours to DNA-damaging agents like CT and RT. Due to its unique mechanism of action and its ability to synergise with a variety of DNA-damaging agents, AsiDNA presents a broad spectrum of potential benefits in the treatment of cancer and justifies further evaluation in combination.

DRIIV-1 study is currently ongoing with an extension Phase (1b) with AsiDNA IV at 600 mg in combination with carboplatin with or without paclitaxel in patients with advanced solid tumours who are eligible to carboplatin alone (Part B1) or carboplatin plus paclitaxel (Part B2) (EudraCT No. 2017-000088-34). Recruitment is ongoing with additional new patients to define the recommended Phase 2 dose (R2PD) of the combination and to further assess the safety, efficacy and PK profile and to collect more data on biomarkers of activity of AsiDNA.

## Conclusion

Administration of AsiDNA by IV route as single agent in patients with advanced solid tumours is safe. The MTD was not reached up to 1300 mg. Biological activity was evidenced by the increase of γH2AX and pHSP90. The dose of 600 mg has been identified as the optimal biological dose for further development given the favourable safety and PK profiles and robust target engagement at this dose.

## Supplementary information

Supplementary Table 1

## Data Availability

Anonymised data set may be available from the corresponding author on request.
