# Supplementary material for: A Phase 1 dose-escalation study to evaluate safety, pharmacokinetics and pharmacodynamics of AsiDNA, a first-in-class DNA repair inhibitor, administered intravenously in patients with advanced solid tumours
Source: Br J Cancer. 2020 Aug 25;123(10):1481–9. doi: 10.1038/s41416-020-01028-8 (PMC7653034; doi:10.1038/s41416-020-01028-8)
Supplement: Supplementary file 1 — Supplementary Table 1 [file 41416_2020_1028_MOESM1_ESM.docx]

**Supplementary Table 1: Analysis of AsiDNA activity biomarkers in tumour tissue and tumour cell proliferation marker signals after one cycle of AsiDNA IV treatment**

| **Dose (mg)** | **Patient ID** | γ**H2AX (fold change from basal level)** | **pHsp90 (fold change from basal level)** | **Ki67 (fold change from basal level)** |
| --- | --- | --- | --- | --- |
| 400 | 001-06 | 0.2 | 30 | 0.71 |
| 400 | 001-09 | 1.33 | 9 | 0.8 |
| 600 | 002-02 | 4 | 1.14 | 0.58 |
| 600 | 003-01 | 20 | 13.33 | 2 |
| 900 | 001-10 | 3 | 2 | 1 |
| 900 | 002-03 | 0.75 | 15 | 1.13 |
| 900 | 003-02 | 1 | 1 | 0.57 |
| 900 | 004-02 | 1.63 | 0.5 | 0.71 |
| 1300 | 001-11 | 2.00 | 1.00 | 1.14 |
| 1300 | 002-04 | 0.82 | 0.20 | 0.83 |
